# Topography of Extracellular Matrix Mediates Vascular Morphogenesis and Migration Speeds in Angiogenesis

**DOI:** 10.1371/journal.pcbi.1000445

**Published:** 2009-07-24

**Authors:** Amy L. Bauer, Trachette L. Jackson, Yi Jiang

**Affiliations:** 1 Theoretical Division, Los Alamos National Laboratory, Los Alamos, New Mexico, United States of America; 2 Department of Mathematics, University of Michigan, Ann Arbor, Michigan, United States of America; The University of Kansas, United States of America

## Abstract

The extracellular matrix plays a critical role in orchestrating the events necessary for wound healing, muscle repair, morphogenesis, new blood vessel growth, and cancer invasion. In this study, we investigate the influence of extracellular matrix topography on the coordination of multi-cellular interactions in the context of angiogenesis. To do this, we validate our spatio-temporal mathematical model of angiogenesis against empirical data, and within this framework, we vary the density of the matrix fibers to simulate different tissue environments and to explore the possibility of manipulating the extracellular matrix to achieve pro- and anti-angiogenic effects. The model predicts specific ranges of matrix fiber densities that maximize sprout extension speed, induce branching, or interrupt normal angiogenesis, which are independently confirmed by experiment. We then explore matrix fiber alignment as a key factor contributing to peak sprout velocities and in mediating cell shape and orientation. We also quantify the effects of proteolytic matrix degradation by the tip cell on sprout velocity and demonstrate that degradation promotes sprout growth at high matrix densities, but has an inhibitory effect at lower densities. Our results are discussed in the context of ECM targeted pro- and anti-angiogenic therapies that can be tested empirically.

## Author Summary

A cell migrating in the extracellular matrix environment has to pull on the matrix fibers to move. When the matrix is too dense, the cell secretes enzymes to degrade the matrix proteins in order to get through. And when the matrix is too sparse, the cell produces matrix proteins to locally increase the “foothold”. How cells interact with the extracellular matrix is important in many processes from wound healing to cancer invasion. We use a computational model to investigate the topography of the matrix on cell migration and coordination in the context of tumor induced new blood vessel growth. The model shows that the density of the matrix fibers can have a strong effect on the extension speed and the morphology of a new blood vessel. Further results show that matrix degradation by the cells can enhance vessel sprout extension at high matrix density, but impede sprout extension at low matrix density. These results can potentially point to new targets for pro- and anti-angiogenesis therapies.

## Introduction

The extracellular matrix (ECM) is a major component of the extravascular tissue region, or stroma, and plays a central role in morphogenesis, including embryogenesis [1], tissue repair and wound healing [2], new blood vessel growth [3], and cancer invasion [4]. A large body of research is concentrated on understanding how cell-ECM interactions impact and regulate morphogenic processes. Results from such investigations illuminate the active role of the ECM in transmitting biochemical signals and mechanical forces that mediate cell survival, phenotype, shape, and orientation. This area continues to be a target of intense investigation.

Cells are equipped with and can upregulate transmembrane receptors that enable them to receive signals from and interact with their environment. Integrins are one such receptor and are stimulated by the various proteins of the ECM [5],[6]. Endothelial cells attach directly to the collagen fibers in the ECM through the 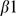 integrin receptors [7]. Biochemical signals originating within the cell can affect integrin-ligand binding affinity and consequently modulate cellular adhesion to the matrix. Focal adhesion complexes form and bind directly to the cell's cytoskeleton [8]. Once assembled, a focal adhesion anchors the cell to the ECM, which is used by the cell for movement. These focal adhesions are assembled and disassembled dynamically to facilitate cell migration. Migratory guidance via focal adhesion binding sites in the ECM is a phenomenon referred to as contact guidance and plays a key role in guiding new vessel growth [9].

### Mechanical properties of the ECM mediate morphogenesis

The physical properties of the ECM, such as density, heterogeneity, and stiffness, that affect cell behavior is also an area of current investigation. Matrigel, a popular gelatinous protein substrate for *in vitro* experiments of angiogenesis, is largely composed of collagen and laminin and contains growth factors, all of which provide an environment conducive to cell survival. In experiments of endothelial cells on Matrigel, increasing the stiffness of the gel or disrupting the organization of the cellular cytoskeleton, inhibits the formation of vascular cell networks [10],[11]. Cells respond to alterations in the mechanical properties of the ECM, for example, by upregulating their focal adhesions on stiffer substrates [12]. For anchorage-dependent cells, including endothelial cells, increasing the stiffness of the ECM therefore results in increased cell traction and slower migration speeds [12]. Measurements of Matrigel stiffness as a function of density show a positive relationship between these two mechanical properties [13]. That is, as density increases, so does matrix stiffness. In light of these two findings, it is not surprising that this experimental study also shows slower cell migration speeds as matrix density increases [13]. Moreover, matrices with higher fiber density transfer less strain to the cell [14] and experiments of endothelial cells cultured on collagen gels demonstrate that directional sprouting, called branching, is induced by collagen matrix tension [15]. Thus, via integrin receptors, the mechanical properties of the ECM influence cell-matrix interactions and modulate cell shape, cell migration speed, and the formation of vascular networks.

Understanding how individual cells interpret biochemical and mechanical signals from the ECM is only a part of the whole picture. Morphogenic processes also require multicellular coordination. In addition to the guidance cues cells receive from the ECM, they also receive signals from each other. During new vessel growth, cells adhere to each other through cell-cell junctions, called cadherins, and in order to migrate, cells must coordinate integrin mediated focal adhesions with these cell-cell bonds. This process is referred to as collective or cluster migration [16]. During collective migration, cell clusters often organize as two-dimensional sheets [16].

Cells also have the ability to condition the ECM for invasion by producing proteolytic enzymes that degrade specific ECM proteins [17]. In addition, cells can synthesize ECM components, such as collagen and fibronectin [11],[18], and can further reorganize the ECM by the forces they exert on it during migration [10],[11],[14]. Collagen fibrils align in response to mechanical loading and cells reorient in the direction of the applied load [14]. Tractional forces exerted by vascular endothelial cells on Matrigel cause cords or tracks of aligned fibers to form promoting cell elongation and motility [11]. As more experimental data are amassed, the ECM is emerging as the vital component to morphogenic processes.

In this work, we extend our cellular model of angiogenesis [19] and validate it against empirical measurements of sprout extension speeds. We then use our model to investigate the effect of ECM topography on vascular morphogenesis and focus on mechanisms controlling cell shape and orientation, sprout extension speeds, and sprout morphology. We show the dependence of sprout extension speed and morphology on matrix density, fiber network connectedness, and fiber orientation. Notably, we observe that varying matrix fiber density affects the likelihood of capillary sprout branching. The model predicts an optimal density for capillary network formation and suggests matrix heterogeneity as a mechanism for sprout branching. We also identify unique ranges of matrix density that promote sprout extension or that interrupt normal angiogenesis, and show that maximal sprout extension speeds are achieved within a density range similar to the density of collagen found in the cornea. Finally, we quantify the effects of proteolytic matrix degradation by the tip cell on sprout velocity and demonstrate that degradation promotes sprout growth at high densities, but has an inhibitory effect at lower densities. Based on these findings, we suggest and discuss several ECM targeted pro- and anti-angiogenesis therapies that can be tested empirically.

## Methods

### Cellular model of angiogenesis

We previously published a cell-based model of tumor-induced angiogenesis that captures endothelial cell migration, growth, and division at the level of individual cells [19]. That model also describes key cell-cell and cell-matrix interactions, including intercellular adhesion, cellular adhesion to matrix components, and chemotaxis to simulate the early events in new capillary sprout formation. In the present study, we extend that model to incorporate additional mechanisms for cellular motility and sprout extension, and use vascular morphogenesis as a framework to study how ECM topography influences intercellular and cell-matrix interactions.

The model is two-dimensional. It uses a lattice-based cellular Potts model describing individual cellular interactions coupled with a partial differential equation to describe the spatio-temporal dynamics of vascular endothelial growth factor. At every time step, the discrete and continuous models feedback on each other, and describe the time evolution of the extravascular tissue space and the developing sprout. The cellular Potts model evolves by the Metropolis algorithm: lattice updates are accepted probabilistically to reduce the total energy of the system in time. The probability of accepting a lattice update is given by
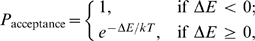
where 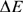 is the change in total energy of the system as a result of the update, 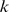 is the Boltzmann constant, and 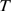 is the effective temperature corresponding to the amplitude of cell membrane fluctuations. A higher temperature corresponds to larger cell membrane fluctuation amplitudes. The energy, 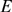, includes a term describing cell-cell and cell-matrix adhesion, a constraint controlling cellular growth, an effective chemotaxis potential, and a continuity constraint. Mathematically, total energy is given by:
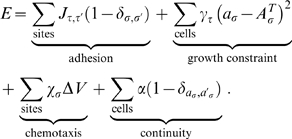
(1)In the first term of Eq. 1, 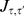 represents the binding energy between model constituents. For example, 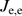 describes the relative strength of cell-cell adhesion that occurs via transmembrane cadherin proteins. Similarly, 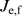 is a measure of the binding affinity between an endothelial cell and a matrix fiber through cell surface integrin receptors. Each endothelial cell is associated with a unique identifying number, 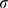. 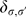 is the Kronecker delta function and 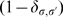 ensures that adhesive energy only accrues at cell surfaces. The second term in Eq. 1 describes the energy expenditure required for cell growth and deformation. Membrane elasticity is described by 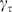, 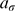 denotes cell 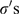 current volume, and 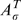 is a specified target volume. For proliferating cells, the target volume is double the initial volume. This growth constraint delivers a penalty to total energy for any deviation from the target volume. In the third term, the parameter 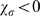 is the effective chemical potential and influences the strength of chemotaxis relative to other parameters in the model. This chemotaxis potential varies depending on cell phenotype (discussed below) and is proportional to the local VEGF gradient, 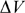, where 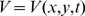 denotes the concentration of VEGF. Cells must simultaneously integrate multiple external stimuli, namely intercellular adhesion, chemotactic incentives, and adherence to extracellular matrix fibers. To do so, endothelial cells deform their shape and dynamically regulate adhesive bonds. In the model, however, it is possible that collectively these external stimuli may cause a cell to be pulled or split in two. To prevent non-biological fragmentation of cells, we introduce a continuity constraint that preserves the physical integrity of each individual cell. This constraint expresses that it is energetically expensive to compromise the physical integrity of a cell and is incorporated into the equation for total energy (Eq. 1) in the last term, where 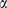 is a continuity constraint that represents the effects of the cytoskeletal matrix of a cell. 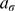 is the current size of the endothelial cell with identifying number 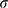, and 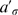 is a breadth first search count of the number of continuous lattice sites occupied by that endothelial cell. Thus, 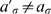 signals that the physical integrity of the cell has been compromised and a penalty to total energy is incurred. Cooperatively, the continuity constraint and the volume constraint implicitly describe the interactions holding the cell together.

**Figure 1:**
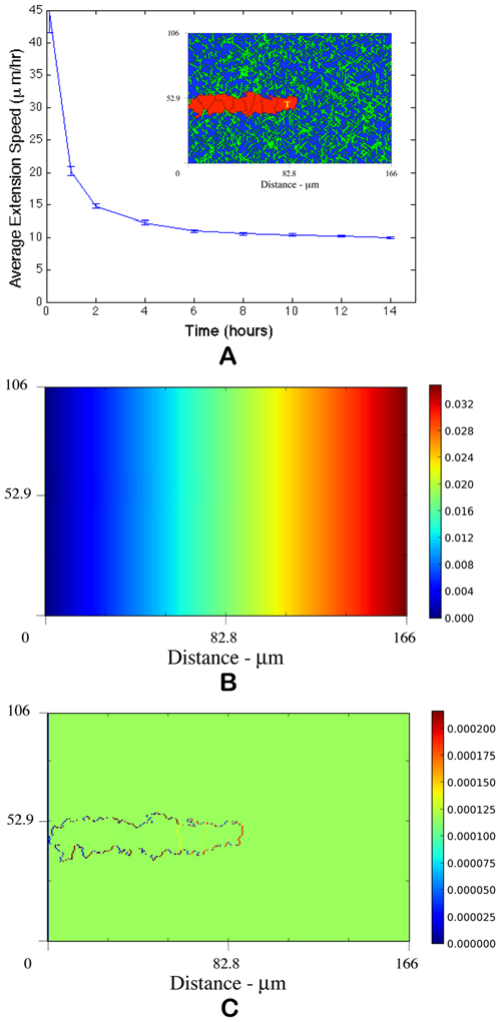
Model validation and geometry. (A) The average extension speeds of our simulated sprouts agree with empirical measurements [26],[39]. Parameters were chosen to maximize sprout extension speeds. Reported speeds are an average of 10 independent simulations using the same parameter set. Error bars represent the standard error from the mean. The inset shows the two-dimensional 166 µm×106 µm geometry of the computational domain and simulated sprout development. Endothelial cells (red) migrate into the domain from a parent blood vessel (left boundary); a line source of VEGF diffuses from a tumor at the right boundary. The space between represents the stroma and is composed of extracellular matrix fibers (green) and interstitial fluid (blue). The sprout tip cell is identified with a T. (B) VEGF concentration profile (pg) showing higher concentrations of VEGF as the cells approach the tumor. (C) VEGF gradient profile (pg) is a better indicator of local VEGF heterogeneities. This image shows larger gradients in the proximity of the tip cell and along the leading edges of the new sprout. Snapshots at 7.8 hours.

The amount of VEGF available at the right hand boundary of the domain is estimated by assuming that in response to a hypoxic environment, quiescent tumor cells secrete a constant amount of VEGF and that VEGF decays at a constant rate. It is reasonable to assume that the concentration of VEGF within the tumor has reached a steady state and therefore that a constant amount of VEGF, denoted 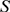, is available at the boundary of the tumor. We use constant boundary conditions for the left (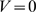) and right 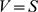 boundaries and periodic boundary conditions in the y-direction. A gradient of VEGF is established as VEGF diffuses through the stroma with constant diffusivity coefficient 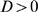, decays at a constant rate 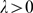, and is bound by endothelial cells, 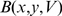. A complete description of the biochemical derivation of the function for endothelial cell binding and uptake of VEGF (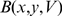) has been previously published [19]. For more direct comparison to other mathematical models of angiogenesis models and to isolate the effects of ECM topology on vessel morphology, we assume that the diffusion coefficient for VEGF in tissue is constant. This is a simplification, however, because the ECM is not homogeneous and VEGF can be bound to and stored in the ECM. Realistically, the diffusion coefficient (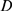) for VEGF in the ECM depends on both space and time. We address the implications of this assumption in the Discussion. Under these assumptions, the concentration profile of VEGF satisfies a partial differential equation of the form:
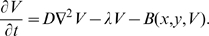
(2)The inset in Figure 1A provides an illustration of the 166 µm×106 µm domain geometry. We initialize the simulation by establishing the steady state solution to Eq. 2. The activation and aggregation of endothelial cells, and subsequent breakdown of basement membrane in response to VEGF [20] is a pre-condition (boundary condition) to the simulation. The breakdown of basement membrane allows endothelial cells to enter the extravascular space through a new vessel opening. Our simulation starts with a single activated endothelial cell ∼10 µm in diameter that has budded from the parent vessel located adjacent to the left hand boundary [20]. We use 10 µm only as an initial estimate of endothelial cell size [21],[22]. Once the simulation begins, the cells immediately deform in shape and elongate. During the simulation, the VEGF field is updated iteratively with cell uptake information, for example as shown in Figure 1B,C. VEGF data is processed by the cells at the cell membrane and incorporated into the model through the chemotaxis term in Eq. 1. From the parent blood vessel, endothelial cells (red) migrate into the domain in response to VEGF that is supplied from a tumor located adjacent to the right hand boundary. The space between represents the stroma and is composed of extracellular matrix fibers (green) and interstitial fluid (blue). The physical meanings of all symbols and their parameter values are summarized in Table 1.

**Table 1:** Table of parameters, which unless otherwise specified, are used for all simulations.

Parameter	Symbol	Model Value	Range	Reference
VEGF Diffusion	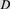	3.6×10^−4^ cm^2^/h		[58]
VEGF Decay	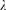	.6498 h^−1^		[58]
VEGF Uptake	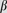	.06 pg/EC/hr		[55],[59],[60]
VEGF Source	S	.035 pg/pixel		[61],[62]
Activation Threshold	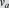	.0001 pg	fixed	[19],[20]
Adhesion		E/L		
*EC–EC*	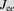	30	[10, 50]	[37]
*EC–Fluid*	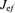	76	I	est
*EC–Matrix*	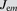	66	[46, 76]	[38]
*Fluid–Fluid*	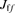	71	I	est
*Fluid–Matrix*	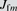	85	I	est
*Matrix–Matrix*	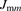	85	I	est
Membrane Elasticity		E/L^4^		
*EC*	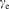	0.8	[0.3, 3]	[32]
*Matrix*	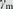	0.5	I	[34]
*Fluid*	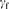	0.5	I	[36]
Chemotactic Sensitivity	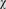	1.11·10^6^ E/conc	[10^4^, 10^7^]	
*Tip Cell*		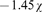		[23]
*Stalk Cell*		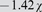		[23]
*Proliferating Cell*		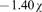		est
Intracellular Continuity	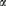	300 E/L	fixed	est
Boltzmann Temperature	kT	2.5 E	[0.25,11]	est

The relative value of the cellular Potts model parameters corresponds to referenced physiological measurements and gives rise to cell behavior observed experimentally. Dimensions are given in terms of length, L, and energy, E. EC denotes endothelial cell, ‘est’ indicates an estimated parameter, and I is an insensitive parameter.

To more accurately capture the cell-cell and cell-matrix interactions that occur during morphogenesis, we implement several additional features to this model. One improvement is the implementation of stalk cell chemotaxis. Stalk cells are not inert, but actively respond to chemotactic signals [23]. As a consequence, cells now migrate as a collective body, a phenomenon called collective or cohort migration [24]. This modification, however, also makes it possible for individual cells, as well as the entire sprout body, to migrate away from the parent vessel, making it necessary to consider cell recruitment from the parent vessel. Cell recruitment is another added feature.

During the early stages of angiogenesis, cells are recruited from the parent vessel to facilitate sprout extension [20],[25]. Kearney et al. [26] measured the number and location of cell divisions that occur over 3.6 hours in *in vitro* vessels 8 days old (a detailed description of these experiments is provided in our discussion of model validation). In these experiments, the sprout field is defined as the area of the parent vessel wall that ultimately gives rise to the new sprout and the sprout itself. The sprout field is further broken down into regions based on distance from the parent vessel and these regions are classified as distal, proximal, and nascent. The authors report that 90% of all cell divisions occur in the parent vessel and the remaining 10% occur at or near the base of the sprout in the nascent area of the sprout field. On average, total proliferation accounts for approximately 5 new cells in 3.6 hours, or 20 cells in 14 hours. This data suggests that there is significant and sufficient proliferation in the primary vessel to account for and facilitate *initial* sprout extension. This data does not suggest that proliferation in other areas of the sprout field does not occur at other times. In fact, it has been established that a new sprout can migrate only a finite distance into the stroma without proliferation and that proliferation is necessary for continued sprout extension [25]. We model sprout extension through a cell-cell adhesion dependent recruitment of additional endothelial cells from the parent vessel. As an endothelial cell at the base of the sprout moves into the stroma, cell-cell adhesion pulls a cell from the parent vessel along with it. In practice, a new cell is added to the base of the sprout when and where the previous cell detaches from the parent vessel wall (left boundary of the simulation domain). We assume, based on the data presented in [26], that there is sufficient proliferation in the parent vessel to provide the additional cells required for initial sprout extension while maintaining the physical integrity of the parent vessel.

As in our previous model, once a cell senses a threshold concentration of VEGF, given by 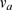, it becomes activated. We recognize that cells have distinct phenotypes that dictate their predominate behavior. Thus, we distinguish between tip cells, cells that are proliferating, and non-proliferating but migrating stalk cells. Tip cells are functionally specialized cells that concentrate their internal cellular machinery to promote motility [23]. Tip cells are highly migratory pathfinding cells and do not proliferate [25],[26]. To model the highly motile nature of the tip cell, we assign it the highest chemotactic coefficient, 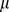. The remainder of the cells are designated as stalk cells and use adhesive binding to and release from the matrix fibers for support and to facilitate cohort migration. Stalk cells also sense chemical gradients but are not highly motile phenotypes. Thus, the stalk cells in the model are assigned a lower, that is weaker, chemotactic coefficient than the specialized tip cell. Proliferating cells are located behind the sprout tip [23],[26] and increase in size as they move through an 18 hour cell cycle clock in preparation for cell division [27]. Cells that are proliferating can still migrate [26]; it is only during the final stage of the cell cycle that endothelial cells stop moving and round up for mitosis (personal communication with C. Little). As we assume that the presence of VEGF increases cell survivability, we do not model endothelial cell apoptosis.

As described in our previous work [19], we model the mesh-like anisotropic structure of the extracellular matrix by randomly distributing 1.1 µm thick bundles of individual collagen fibrils at random discrete orientations between −90 and 90 degrees. Unless otherwise stated, model matrix fibers comprise approximately 40% of the total stroma and the distribution of the ECM is heterogeneous, with regions of varying densities as can be seen in Figure 1A and Figure 7D. The cells move on top of the 2D ECM model and interact with the matrix fibers at the cell membrane through the adhesion term in Eq. 1. To relate the density (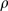) of this model fibrillar matrix to physiological values, we measure matrix fiber density as the ratio of the interstitium occupied by matrix molecules to total tissue space, 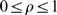, and compare it to measured values of the volume fraction of collagen fibers in healthy tissues [28]. In order to isolate and control the effects of the matrix topology on cellular behavior and sprout morphology we look at a static ECM, that is we do not model ECM rearrangement or dynamic matrix fiber cross-linking and stiffness. We do, however, consider endothelial cell matrix degradation in a series of studies presented in Results.

No single model has been proposed that incorporates every aspect of all processes involved in sprouting angiogenesis, nor is this level of complexity necessary for a model to be useful or predictive. It is not our intention to include every bio-chemical or mechanical dynamic at play during angiogenesis. We develop this two-dimensional cell-based model as a step towards elucidating cellular level dynamics fundamental to angiogenesis, including cell growth and migration, and cell-cell and cell-matrix interactions. Consequently, we do not incorporate processes or dynamics at the intracellular level. For example, we describe endothelial cell binding of VEGF to determine cell activation and to capture local variations in VEGF gradients, but neglect intracellular molecular pathways signaled downstream of the receptor-ligand complex. Moreover, our focus is on early angiogenic events and therefore we also do not consider the effects of blood flow on remodeling of mature vascular beds. Numerical studies of flow-induced vascular remodeling have been given attention in McDougall et al. [29], and Pries and Secomb [30],[31].

### Parameter calibration

As is the case in many other simulations of biological systems, when we do not have direct experimental measurements for all of the parameters, choosing these parameter values is not trivial. A list of values and references for our model parameters is provided in Table 1. A parameter is derived from experimental data whenever possible, otherwise it is estimated and denoted ‘est’. Fortunately, a sensitivity analysis (discussed later) shows that the dynamics of our model are quite robust to substantial variations in some parameters and tells us exactly which parameters are most critical. We can then choose from a range of parameter values that exhibits the general class of behavior consistent with experimental observations. See Table 1 for these parameter ranges and Table 3 for the effect of parameter perturbations, as well as, supplemental Figures S1 and S2 for examples of cellular behavior under different parameter sets. In the cellular Potts model, the relative value, not the absolute value, of the parameters corresponds to available physiological measurements and gives rise to a cell behavior observed experimentally. For example, the Young's modulus for human vascular endothelial cells is estimated at 2.01*10^5^ Pa [32]. The Young's modulus of a collagen fiber in aqueous conditions is between 0.2–0.8 GPa [33]. However, the modulus of a collagen gel network is much lower and is measured at 7.5 Pa [34]. Although interstitial fluid compressibility (water) is estimated to be 2.2 GPa [35], indicating it's hard to compress under uniform pressure, it deforms easily, that is, the shear modulus is low and is measured at 10^−6^ Pa [36]. The qualitative parameters corresponding to these quantitative measurements are 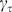 where 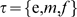. Thus, the elastic modulus of endothelial cells>matrix fibers>interstitial fluid (0.2 MPa>7.5 Pa>10^−6^ Pa) and is reflected in the relative values of the corresponding parameters 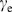, 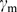, and 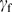. In a similar manner, the coupling parameters, 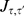, describe the relative adhesion strengths among endothelial cells, matrix fibers, and interstitial fluid. For instance, choosing 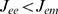 reflects that fact that endothelial cells have a higher binding affinity to each other, via cadherin receptors and gap junctions for example, than they do to matrix fibers [37],[38]. The chemotactic potential, 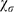, is chosen so that its contribution to the change in total energy is the same order of magnitude as the contribution to total energy from adhesion or growth. The difference between the concentration of VEGF at two adjacent lattice sites is on the order of 10^−4^. Therefore, to balance adhesion and growth, 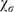 must be on the order of 10^6^. We calibrate this parameter to maximize sprout extension speeds. Similarly, the parameter for continuity, 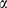, is chosen so that cells will not dissociate. This is achieved by setting 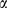 greater than the collective contribution to total energy from the other terms. By equating the time it takes an endothelial cell to divide during the simulation with the endothelial cell cycle duration of 18 hours, we convert Monte Carlo steps to real time units. In the simulations reported in this paper, 1 Monte Carlo step is equivalent to 1 minute. Since this model has several enhancements over the previous model [19], there are a different number of parameters, which necessitates recalibration of all the parameters. Therefore, some parameters take on different values.

## Results

### Model validation

The canonical benchmark for validating models of tumor-induced angiogenesis is the rabbit cornea assay [39],[40]. In this *in vivo* experimental model, tumor implants are placed in a corneal pocket approximately 1–2 mm from the limbus. New vessel growth is measured with an ocular micrometer at 10×, which has a measurement error of ±0.1 mm or 100 µm. Initially, growth is linear and sprout extension speeds are estimated at a rate of 0.5 mm/day, or 20.8±4.2 µm/hr. Sprouts then progress at average speeds estimated to be between 0.25–0.50 mm/day, or 10.4–20.8±4.2 µm/hr. More recent measurements of sprout extension speeds during angiogenesis are reported in Kearney et al. [26]. In this study, embryonic stem cells containing an enhanced green fluorescent protein are differentiated *in vitro* to form primitive vessels. Day 8 cell cultures are imaged within an ∼160 µm^2^ area at 1 minute intervals for 10 hours and show sprouting angiogenesis over this period. The average extension speed for newly formed sprouts is 14 µm/hr and ranges from 5 to 27 µm/hr. For cell survival, growth factor is present and is qualitatively characterized as providing a diffuse, or shallow, gradient. No quantitative data pertaining to growth factor gradients or the effect of chemotaxis during vessel growth are reported [26].

We use the above experimental models and reported extension speeds as a close approximation to our model of *in vivo* angiogenesis for quantitative comparison and validation. We simulate new sprout formation originating from a parent vessel in the presence of a diffusible VEGF field, which creates a shallow VEGF gradient. We measure average extension speeds over a 14 hour period in a domain 100 µm by 160 µm. As was done in Kearney et al. [26], we calculate average sprout velocities as total sprout tip displacement in time and measure this displacement as the distance from the base of the new sprout to the sprout tip. Figure 1A shows average sprout extension speed over time for our simulated sprouts. Reported speeds are an average of at least 10 independent simulations using the same initial VEGF profile and parameter set as given in Table 1. Error bars represent the standard error from the mean. The average extension speeds of our simulated sprouts are within the ranges of average sprout speeds measured by both Kearney et al. [26] and Gimbrone et al. [39]. Table 2 summarizes various morphological measurements for the simulated sprouts. It shows that the average velocity, thickness, and cell size of the simulated sprouts compare favorably to relevant experimental measurements. Sprout velocity is given at 10 hours for direct comparison to [26] and averaged over 14 hours. Sprout thicknesses and cell size are within normal physiological ranges. There are many different cell shapes and sizes and vessel morphologies, however, that can be obtained *in vivo* and *in vitro* given different environmental factors (VEGF profile, ECM topology and stiffness, inhibitory factors, other cell types, etc.). In this manuscript, we investigate several of these dependencies and as we discuss below specific model parameters can be tuned to reproduce different cellular interactions and environments.

**Table 2:** A comparison of various morphological measurements for the simulated sprouts showing average velocity, thickness, and cell size compare favorably to experimental measurements.

	Simulated	Observed	Reference
Velocity	10.4±.2 µm/hr	14 µm/hr (at 10 h)	[26]
	16.0±.6 µm/hr	10.4–20.8±4.2 µm/hr	[39]
Thickness	16.2±2.4 µm	15 µm	[41]
		17±4 µm	[pc]
Cell Size	15–40 µm	20–40 µm	[23],[26],[49],[63]

Sprout velocity is given at 10 hours for direct comparison to [26] and also averaged over 14 hours. Sprout thicknesses and cell size are within normal physiological ranges. [pc]: personal communication with S. Heilshorn.

Figure 1A indicates that average sprout extension speed changes as a function of time. Within the first two hours, speeds average ∼30 µm/hr and the new sprout consists of only 1–2 endothelial cells. At two hours, sprouts contain an average of 3 cells, and at 4 hours, there are a total of 5–6 cells. Over time, as more cells are added to the developing sprout, cell-cell adhesion and cellular adhesion to the extracelluar matrix slow the sprout extension speed. The inset in Figure 1A shows the geometry of the computational domain and simulated sprout development at 7.8 hours. As shown, simulated sprouts are approximately one cell diameter wide, which compares quantitatively well to reported VEGF induced vessel diameters [41],[42]. Here and in all simulation snapshots, tip cells are identified with a ‘T’. In moving multicellular clusters, rear retraction is a collective process that involves many cells simultaneously [16]. A natural result of the cell-based model is that cells exhibit rear retraction, which refers to the ability of a cell to release its trailing adhesive bonds with the extracellular matrix during migration. Collective migration, another characteristic dynamic observed during sprout growth, is also evident during the simulations (see videos). The VEGF concentration profile in picograms (pg) at 7.8 hours is given in Figure 1B. Higher concentrations of VEGF are encountered as the cells approach the tumor. However, because cell uptake of VEGF is small compared to the amount of available VEGF, it is difficult to discern the heterogeneities in the VEGF profile from this figure. Figure 1C is the VEGF gradient profile (pg) at 7.8 hours and is a better indicator of the changes in local VEGF concentration. This image shows larger gradients in the proximity of the tip cell and along the leading edges of the new sprout.

On average, simulated sprouts migrate 160 µm and reach the domain boundary in approximately 15.6 hours, before any cells in the sprout complete their cell cycle and proliferate. We do not expect to see proliferation in the new sprout because the simulation duration is less than the 18 hour cell cycle and the cell cycle clock is set to zero for newly recruited cells to simulate the very onset of angiogenesis. In our simulations, sprout extension is facilitated by cell recruitment from the parent vessel. Between 15 and 20 cells are typically recruited, which agrees with the number of cells we estimate would be available for recruitment based on parent vessel cell proliferation reported by Kearney et al. [26]. In those experiments [26], proliferation in the parent vessel was measured for day 8 sprouts, which likely has cells at various stages in their cell cycles. Proliferation in the new sprout is another mechanism for sprout extension. Thus, we consider the possibility that cells recruited from the parent vessel may be in different stages of their cell cycles by initializing the cell cycle clock of each recruited cell at randomly generated times. We observe no differences in extension speeds, sprout morphology, or the number of cells recruited as a result of the assumption we make for cell cycle initialization (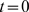 or 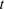 random). This suggests that, in the model, stalk cell proliferation and cell recruitment from the parent vessel are complementary mechanisms for sprout extension.

By adjusting key model parameters, we are able to simulate various morphogenic phenomena. For example, by increasing the chemotactic sensitivity of cells in the sprout stalk and decreasing the parameter controlling cellular adhesion to the matrix, 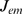, we are able to capture stalk cell migration and translocation along the side of a developing sprout (Video S1). This phenomena, where stalk cells weaken their adhesive bonds to the extracellular matrix and instead use cell-cell adhesion to facilitate rapid migration, frequently occurs in embryogenesis (personal communication with C. Little) and is described as preferential migration to stretched cells [43]. Compare Video S1 with Figure 1(f) in Szabo et al. 2007 [43]. Figure S1 shows the morphology for one particular set of parameter values corresponding to weaker cell-cell and cell-matrix adhesion and stronger chemotaxis. In this simulation, cells elongate to approximately 40 µm in length, fewer cells are recruited from the parent vessel, and the average extension speed at 14 hours slows to 6.8 µm/hr. The length scale is consistent with experimental measurements of endothelial cell elongation [23],[44].

Figure 5 from Oakley *et al.* 1997 shows images from experiments using human fibroblasts stained for actin (e) and tubulin (f) on micro-machined grooved substratum [45]. These experiments demonstrate that cells alter their shape, orientation, and polarity to align with the direction of the grooves (double-headed arrow), exhibiting topographic, or contact, guidance. Figure S2 is a simulation designed to mimic these experiments by isolating the cellular response to topographical guidance on similarly patterned substratum. In this simulation, there is no chemotaxis and no cell-cell contact; cells respond only to topographical cues in the extracellular matrix. Simulated cells alter their shape and orient in the direction of the matrix fibers. Figure S2 bears a striking resemblance to the cell shapes observed in [45]. We are also able to simulate interstitial invasion/migration by a single cell by turning off proliferation and cell recruitment but leaving all other parameters unchanged (Video S2). This simulation is especially relevant in the context of fibroblast recruitment during wound healing and tumor cell invasion (e.g., glioblastoma, the most malignant form of brain cancer [46]), where understanding cell-matrix interactions and directed motility are critical mechanisms for highly motile or invasive cell phenotypes.

### Model predicts ranges of matrix fiber density that may inhibit angiogenesis *in vivo*

We design a set of numerical experiments allowing us to observe the onset of angiogenesis in extravascular environments of varying matrix fiber density. We consider matrix fiber densities given as a fraction of the total interstitial area, 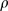. As a measure of matrix orientation equivalency, the total fiber orientation in both the x and the y direction is calculated as we increased the matrix density. The total x and total y fiber orientation do not vary with changes in total matrix density. Besides varying the matrix density, all other parameters are held fixed. All simulations last the same duration corresponding to approximately 14 hours.

**Figure 2:**
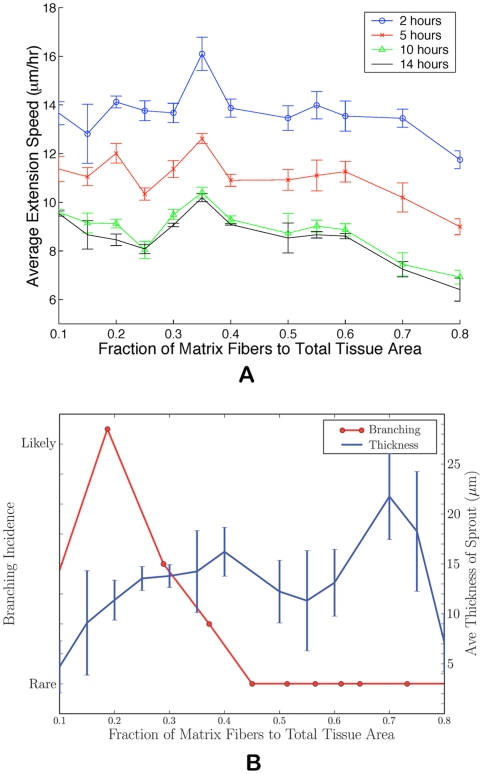
Matrix density influences sprout velocity and branching. (A) Dependence of average sprout extension speed on the density of the extracellular matrix. The model predicts that average extension speeds are maximal in the fiber fraction range 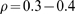. Above 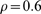, extension speeds are significantly reduced and for 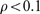 and 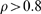 normal angiogenesis is interrupted suggesting that modulating matrix density may be an effective anti-angiogenesis therapy. (B) Quantification of morphological properties of the sprout showing sprout thicknesses within normal physiological ranges but dependent on matrix density and a distinct range of fiber density conductive to branching.

The average rate at which the sprout grows and migrates, or its average extension speed, is calculated as the total tip cell displacement in time. Average extension speeds in microns per hour (µm/hr) versus matrix fiber density (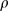) are graphed in Figure 2A at various times (2, 5, 10, 14 hours) during sprout development. We identify qualitative measures to describe and differentiate between various capillary sprout morphologies, such as the thickness of the sprout, its tortuosity, and whether sprout branching or anastomosis occur. Following Kearney et al., we define a sprout branch as one or more cells that extend, or bud, from the primary sprout body at least 10 µm [26]. We report capillary sprout thickness and the incidence of branching versus the fraction of matrix fibers present in the stroma in Figure 2B.

**Figure 3:**
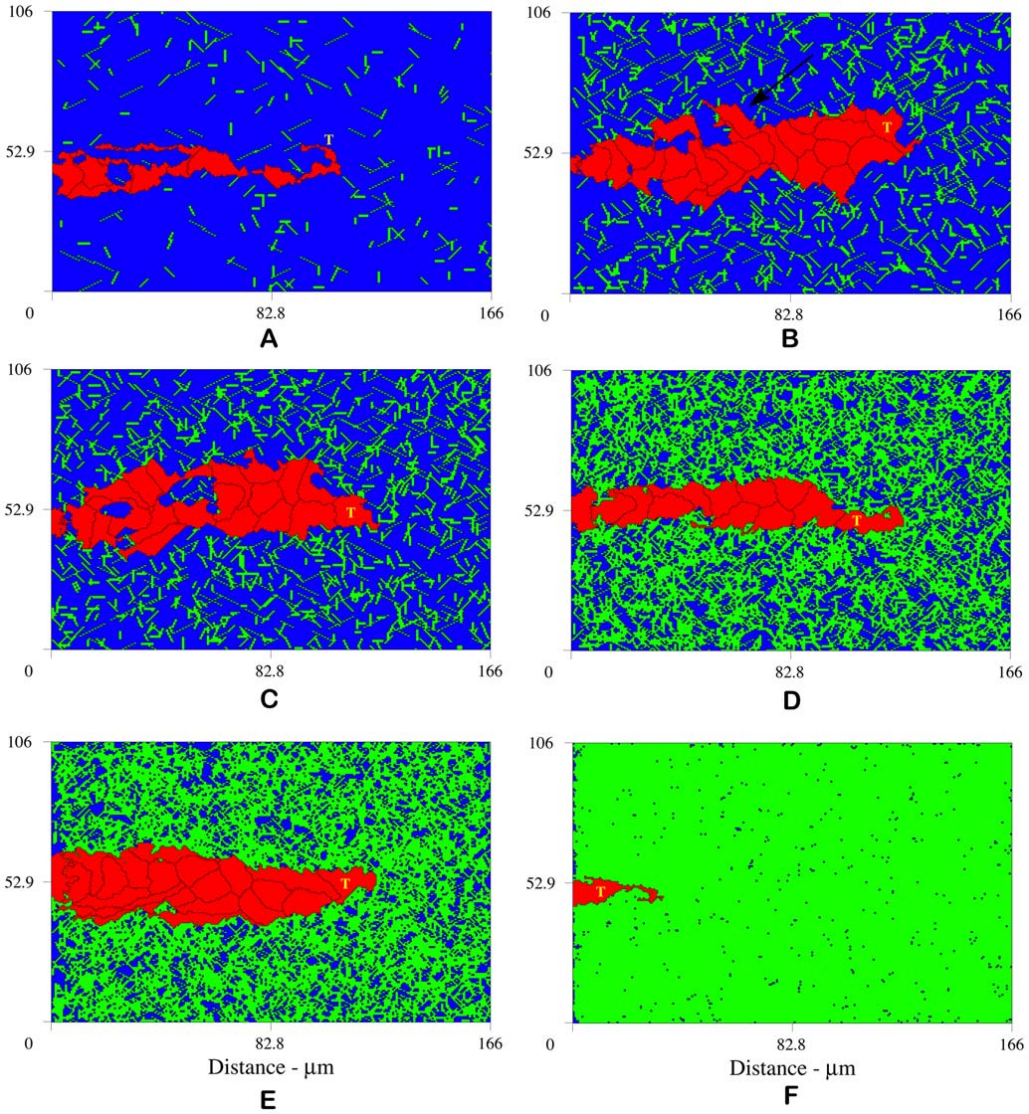
Plots showing the effect of the mechanical properties and heterogeneity of the ECM on sprout morphology and viability. From top left to bottom right: (A) 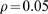, interruption of normal angiogenesis and loss of sprout viability; (B) 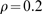, high matrix heterogeneity induces branching (arrow points to new branch); (C) 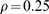, anastomosis; (D) 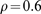, more homogeneous matrix fiber network produces linear sprouts; (E) 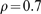, higher matrix homogeneity causes loss of strong guidance cues resulting in wider and slower sprout formation; and (F) 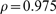, complete inhibition of angiogenesis at high matrix density. Snapshots at 14 hours.

Figure 2 demonstrates that the density of the matrix impacts the average rate at which a capillary sprout extends and the resulting sprout morphology. At very low ratios (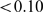), the matrix fibers are sparse, disconnected filaments (Figure 3A). In a study of vasculogenesis using endothelial cells plated on varying densities of collagen or fibronectin, cell attachment, spreading, and tube formation are maximal on dishes of intermediate density, reported to be 100–500 ng/cm^2^ [47]. Whereas, at matrix densities below 100 ng/cm^2^, cells detach from the substrate and lose their viability [47]. Our model predicts a coincident interruption of normal angiogenesis and loss of sprout viability at very low matrix fiber densities (<0.10). Moreover, experimental data shows that matrices with lower fibril density transfer more strain to the cell [14]. We capture the morphological consequences of this relationship by inferring strain rate effects on morphology through changes in matrix density. A simulation of sprout development on a low fiber density matrix can be seen in Figure 3A and shows severe cell elongation at 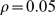. Compare these cells with those shown in the inset of Figure 1A, which is an identical simulation except for an increase in the ECM density (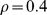). This higher density matrix has an effect similar to that of transferring less strain to the cells and consequently the cells are rounder. Additionally, because there are more focal adhesion sites in this denser matrix, cells are able to maintain their cell-cell contacts and develop as a cohesive body. We do not report migration speeds for 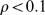 or 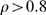 because sprouts show developmental defects, that is, cells are severely elongated, detach from each other, do not grow, or do not form a cohesive sprout body.

For 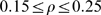, the fiber network is highly inhomogeneous, and we know that lower matrix densities transfer a larger amounts of strain to the cells. As a result we see an increase in cell spreading and a thickening of the new sprout as compared to those morphologies seen for 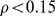 (compare Figures 3A and 3B). These values of 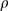 correspond to the same fraction of collagen present in subcutaneous tissue (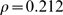) and some skeletal muscle (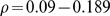) [28]. Figure 2B quantifies the incidence of branching for sprouts developing in different matrix densities. Remarkably, we see a distinct range of densities, 0.20–0.30, where new buds develop from the main sprout body and branches begin to form (see arrow in Figure 3B). This observation suggests that a high degree of fiber heterogeneity, which is related to mechanical mechanisms, such as ECM tension transfer to cells, may promote branching. This observation is consistent with reports that collagen matrix tension induces directional sprouting in endothelial cells [15]. Figure 3C shows sprout development on a matrix where 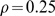. Morphologies that could be interpreted as anastomosis (loop formation) are evident and are only seen at this density. Figure 2A shows (i) a clear range of matrix density that encourages sprout migration and results in faster average speeds and (ii) density ranges that present a physical barrier to migration and inhibit sprout growth and results in slower extension speeds. The peak in the graph at 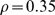 indicates that sprout extension speeds are fastest at intermediate densities between 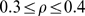 and suggests an optimal matrix density for promoting angiogenesis. For comparison, this range of matrix density is near the physiological fraction of collagen fibers found in the cornea [28]. A possible mechanistic explanation for the existence of a peak extension velocity is that the mechanical properties of the ECM around 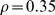 provide contact guidance cues that complement or are aligned with chemotactic gradients. Referring again to Figure 2A, we see that peak migration speeds are prominent at 2 hours, but are still evident, although to a lesser extent, at 10 and 14 hours. Thus, these results do not depend on time. Our finding that maximum migration speeds depend on matrix density is supported by empirical measurements of endothelial cell migration speeds on various fibronectin concentrations (0.5, 1, 5, 20, 40 µg/cm^2^) demonstrating peak migration speeds at intermediate concentrations (5 µg/cm^2^) [48].

As matrix density increases, the network of connected fibers is extensive. Higher fiber density translates into greater matrix homogeneity and a loss of strong guidance cues from fiber heterogeneity. Chemotaxis then plays a stronger role in sprout guidance thereby producing linear sprouts (Figure 3D). Consequently, we do not observe any branching at densities above 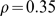. At a fiber density of 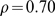, less tension is transferred to the cells. Cells experiencing less tension are rounder. Wider and slower sprouts form at this matrix density (Figure 3E). Above 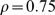, very high matrix densities actually establish a physical barrier to migration and we see a corresponding reduction in sprout extension speed due to increased focal adhesion contacts. Figure 3F shows complete inhibition of angiogenesis at 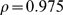 as cell adherence to matrix fibers dominates chemotactic incentives.

Looking at Figures 2B and 3, average sprout thickness is within the observed physiological range of 1–3 cells wide, but does show a dependency on matrix density. For very low densities (A), the cells form a very thin, unstable sprout (<1 cell wide). For intermediate densities (B–D and Figure 1A), sprouts are stable and approximately 10–15 µm wide (1–2 cells). As matrix density increases (E), sprouts thicken and are on average 20–25 µm wide (2–3 cells). As Figure 3F shows, at very high densities, sprouts are unable to form. The results presented in this section were very recently confirmed by experiments performed independently and unbeknownst to us by Prof. Sarah Heilshorn and Amir Shamloo in the Materials Science and Engineering Department at Stanford University (personal communication, manuscript in preparation). In Heilshorn and Shamloo's experiments, sprouting formation from dermal microvascular endothelial cells is studied in different collagen concentrations (0.3, 0.7, 1.2, 1.9, and 2.7 mg/mL) in a microfluidic device (for details on their microfluidic device see [49]). The cells are subjected to equilibrium VEGF concentration gradients of 50 ng/mL/mm (with minimum and maximum VEGF concentrations of 100 and 150 ng/mL at the boundaries of the cell culture chamber) and are incubated for 2–4 days. No sprout formation occurs at 0.3 mg/mL. At low collagen concentrations (0.7 mg/mL), some tracks of cells can be seen to form unstable sprouting structures and sprouts are less than 10 µm wide (compare to Figure 3A). Stable sprouting can be seen at a collagen concentrations of 1.2 and 1.9 mg/mL and sprout are 8±2–18±4 µm thick (compare to Figure 3B–D and inset in Figure 1A). In addition, branching of sprouts is only observed at a collagen concentration of 1.2 mg/mL confirming our finding that branching occurs only in a specific matrix density range. At final collagen concentration of 2.7 mg/mL, sprouts are 45±15 µm thick or do not grow at all (compare to Figure 3E,F). Our model accurately predicts both the qualitative and quantitative relationships between matrix density and sprout thickness and occurrence of branching confirmed by experiment.

### Network connectedness and matrix fiber alignment influence sprout extension speeds

Based on our earlier observations, the density of the ECM affects capillary sprout migration speeds. As matrix density is increased, a connected fibrous network develops which could be a mechanism for differences in observed average speeds. We hypothesized that peak extension speeds occur when the mechanical properties of the ECM provide contact guidance cues that are aligned with the chemotactic gradients. To examine the effects of matrix fiber alignment on average rates of capillary sprout elongation, we devise another set of numerical experiments. If matrix fiber alignment plays a prominent role in sprout migration, we would expect more rapid rates of sprout elongation when matrix fibers are aligned parallel to VEGF gradients than when fibers are aligned perpendicular to the gradient. We look at three specific cases: matrix fibers aligned perpendicular to VEGF gradients, matrix fibers aligned parallel to the VEGF gradient, and a combination of horizontal and vertical fibers only. We compare these test cases with the baseline simulations of sprout development on matrices of random fiber orientation. We distinguish and refer to these three cases by the angle that is formed between the fiber axis and the axis of the VEGF gradient, that is, 0° denotes a matrix with fibers aligned parallel to the gradient and 90° identifies a matrix of fibers perpendicular to the VEGF gradient. These numerical experiments represent a simplified replica of the matrix fiber restructuring and fiber alignment that occurs as a result of the tractional forces exerted by endothelial cells during migration [11],[15]. All matrices have the same matrix fiber density.

As matrix fiber density increases, both the number of focal adhesion binding sites available in the ECM and the connectivity of the fiber network increase. As a measure of connectivity, we consider the network connected if there exists a continuous path along matrix fibers from the parent vessel to the source of chemoattractant. As the density of matrix fibers increases, there will be a density that guarantees network connectedness. This threshold density is known as the percolation threshold. Our model fiber networks are constructed by randomly placing fibers at randomly selected but discrete orientations: 0°, ±30°, ±45°, ±60°, and 90°. Consequently, our fiber network most closely approximates a triangular lattice. We estimate that the percolation threshold in our fiber networks occurs between 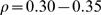. Recall that we define matrix density, 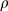, as the fraction of total tissue space occupied by collagen fibers. This can be interpreted as the probability that a matrix fiber occupies, that is, a bond exists between, two neighboring lattice sites. The bond percolation thresholds depend on lattice geometry and is 0.35 for a two-dimensional triangular lattice [50]. The matrix percolation threshold observed in our random matrices corresponds to the bond percolation threshold for a two-dimensional triangular lattice. Interestingly, this percolation threshold is coincident with the density at which our model predicts maximum sprout extension rates. This finding suggests that capillary sprout extension is positively related to the connectedness of the network. We believe that this is because, at the percolation threshold, “tracks” of matrix form, and, near this matrix density, the adhesive and chemotactic energies are well balanced. These factors, in combination, provide strong contact guidance cues to and facilitate the motility of the developing sprout. Referring again to Figure 2A, as the density of the matrix increases above the percolation threshold, sprout extension slows. Thus, even though a connected fiber network is also present at higher densities, higher matrix density is also commensurate with a greater number of focal adhesion binding sites, which impedes cell, and therefore sprout, mobility.

**Figure 4:**
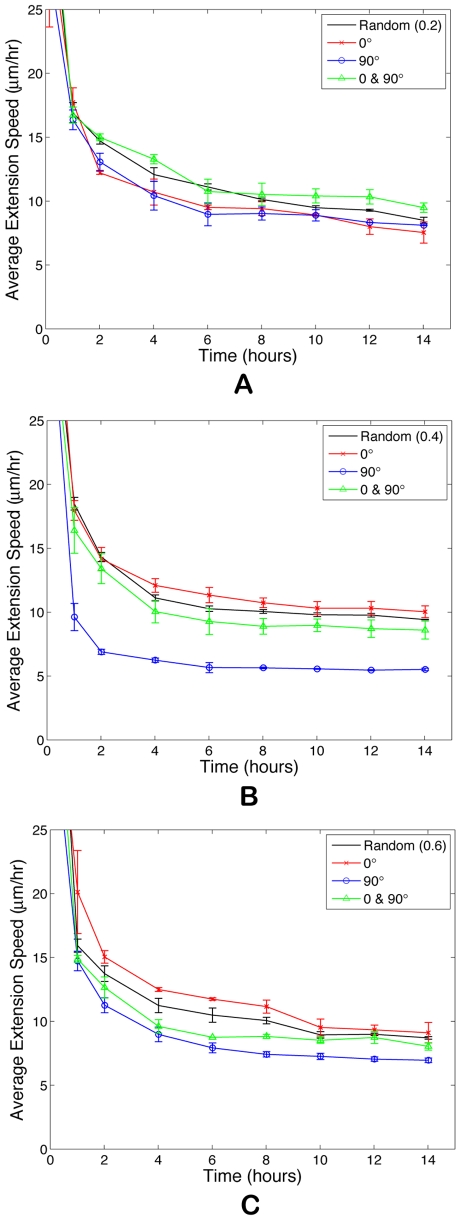
Evidence that mechanical cues, or contact guidance, from the ECM affects sprout extension. (A) At 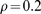, fiber network is not well connected and matrix fiber alignment does not have a strong effect on sprout extension speeds. At 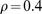 (B) and 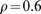 (C), rates of sprout extension are more rapid when matrix fibers are aligned parallel to VEGF gradients (0°) than when matrix fibers are aligned perpendicular to the gradient (90°).

Figure 4A–C reports the average extension speed of new sprouts forming on these restructured matrices for 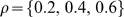 respectively. The baseline for comparison is the average extension speed for sprouts formed on matrices with random fiber alignment and is plotted as a solid black line in each plot. At 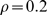, there are fewer focal adhesion sites in the ECM and the matrix fibers do not form a well connected network. Consequently, at this density, matrix fiber alignment does not have a strong effect on sprout extension speeds. At 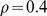 and 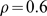, sprouts achieve statistically significant higher average extension speeds when the fibers are aligned parallel to the VEGF gradient (0°) than when fibers are aligned perpendicular to the chemogradient (90°). The slowest speeds occur on matrices with fibers aligned perpendicular to the VEGF gradient. Interestingly, sprout extension speeds on a matrix composed of randomly oriented fibers are almost as fast as those observed on matrices aligned parallel to the gradient (0°). The reason for this is clear if we consider the vector describing net contact guidance cues due to fiber orientation. For strictly 0° or 90° matrices, the net contact guidance cues are in the 0° and 90° directions respectively. For matrices composed of fibers aligned randomly in both 0° and ±90°, the net cue is at a ±45° angle. This explains why 0° matrices facilitate the fastest extension speeds and 90° matrices the slowest. For matrices with completely random fiber orientations, the resultant contact guidance cue is at a ±11° angle. This is calculated by vector summation of the discrete fiber orientations at 0°, ±30°, ±45°, ±60°, 90°: 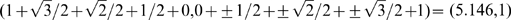. Thus, 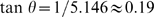, and 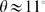. Since the contact guidance cue for random matrices is approximately aligned with the VEGF gradient, this accounts for our observation that the corresponding extension speeds are close to those speeds recorded on 0° matrices. In these computer generated matrices, the fibers are oriented at discrete angles and thus have a net orientation. Biologically, we are not limited to these discrete angles. Depending on the tissue type, fibers may already be aligned, for instance in muscle, or the tissue may be isotropic and lack any structural orientation. Compared to 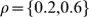, the effect of matrix fiber alignment is greatest at 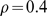. This is because at 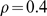, the fiber network is well connected and provides adequate focal adhesion sites, but still retains sufficient anisotropy such that strong guidance cues are transferred through fiber orientation. At higher densities (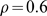), even though there are ample focal adhesion binding sites, the matrix is more homogeneous, matrix “tracks” become less evident, and strong migratory cues from matrix anisotropies are lost. Consequently, the effect of matrix alignment on average extension speed decreases. These results support our hypothesis that when mechanical or contact guidance cues from the ECM are aligned with the direction of cell chemotaxis, these mechanisms for motility cooperate and promote sprout extension.

### Cell shape and orientation are linked to matrix fiber alignment

**Figure 5:**
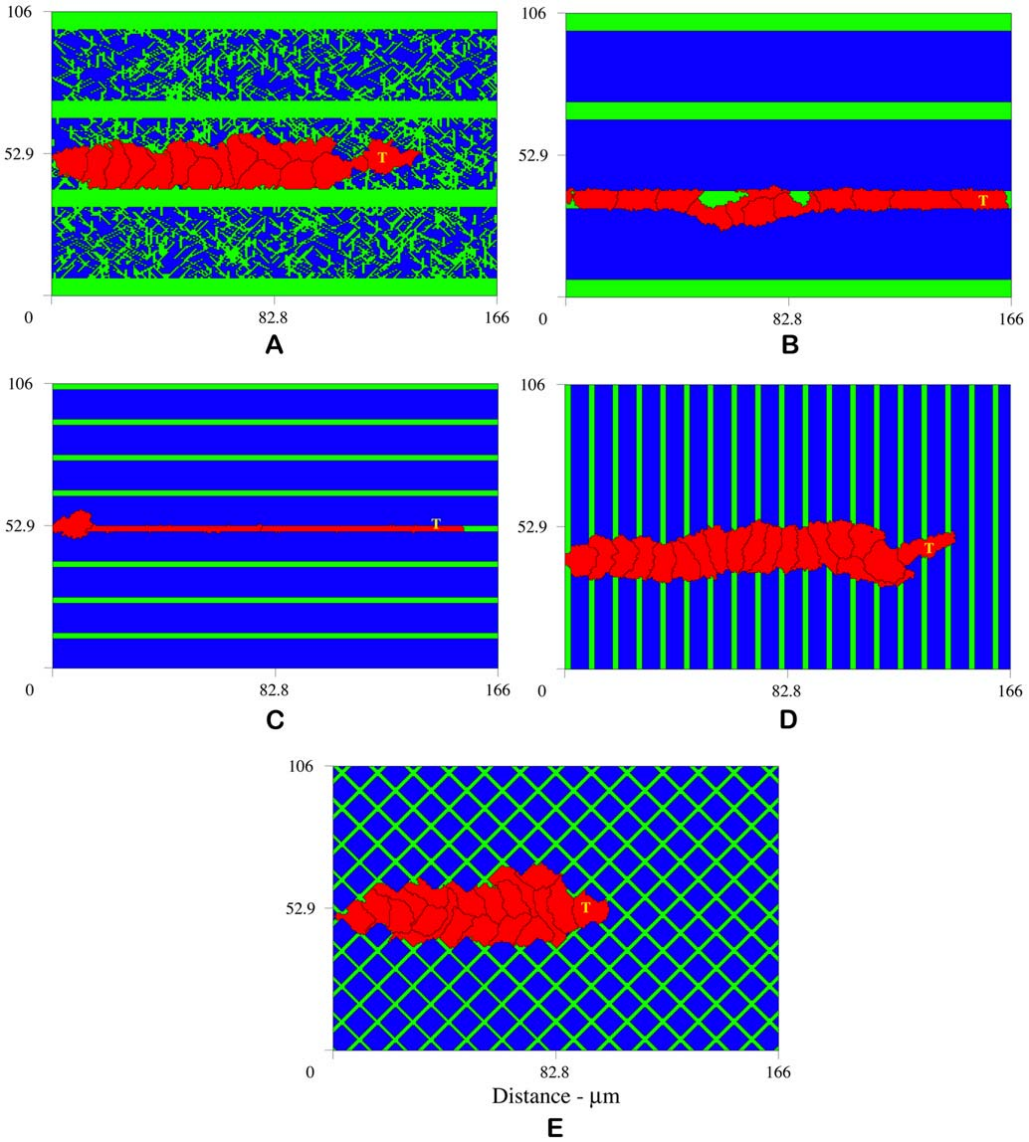
Sprouts developing on patterned matrices reveal a strong correspondence between fiber alignment and cell shape and orientation. Sprouts migrate toward higher concentrations of VEGF, however, cells elongate and are clearly oriented in the direction of the matrix cords. (A) Matrix of randomly distributed fibers augmented with horizontal cords 7.2 µm thick, (B) matrix cords 7.2 µm thick aligned horizontally, (C) horizontal cords 2.2 µm thick, (D) vertical cords 2.2 µm thick, and (E) crosshatched cords. Horizontal cords are aligned with to the VEGF gradient (0°); vertical cords are perpendicular to the gradient (90°); crosshatched cords form a ±45° angle with the VEGF gradient. These results demonstrate the important role of contact guidance and tissue structure in determining cell shape and orientation. Snapshots at 12.5 hours.

In light of the above results, we construct patterned matrix topographies to look at the effect of unambiguous contact guidance cues on cell shape, orientation, and sprout morphology. In these numerical experiments, instead of distributing fiber bundles, we engineer matrix cord patterns that vary in width and orientation. As a baseline, we augment a matrix of randomly distributed fibers with horizontal cords 7.2 µm thick (Figure 5A). Figure 5B–E shows sprout development on matrix cords 7.2 µm thick aligned horizontally, horizontal cords 2.2 µm thick, vertical cords 2.2 µm thick, and crosshatched cords. Horizontal cords are aligned parallel to the VEGF gradient (0°); vertical cords are perpendicular to the gradient (90°); crosshatched cords form a ±45° angle with the gradient. Except for the topography of the ECM, all other model parameters are unchanged.

We find a strong correspondence between fiber alignment and cell shape and orientation. We define cell orientation as the axis of elongation. In Figure 5A, the density of ambient fibers is great enough to form a well connected mesh and facilitate migration, whereas the higher density matrix cords present a physical barrier that requires more energy to overcome. The anisotropy of the fiber mesh promotes variable cell shapes with no obvious cell orientation. In contrast, in the absence of an ambient fiber mesh, cells quickly adhere to the matrix cords (Figure 5B). Cells orient and elongate in the direction of the horizontal cords. Figure 5C shows the result of reducing cord thickness roughly 1/2 cell diameter from 7.2 to 2.2 µm. Cells dramatically elongate and orient in the direction of the VEGF gradient. Compare these two cases to Figure 5A and notice that thinner more linear sprouts develop when strong and unambiguous contact guidance cues are aligned in the direction of chemotaxis. Next we examine the effects of matrix cords aligned perpendicular to the gradient. The results are shown in Figure 5D. In this case, although the sprout migrates toward higher concentrations of VEGF, cells elongate and clearly orient in the direction of the matrix cords, perpendicular to the gradient. Figure 5E depicts sprout formation on crosshatched matrix topography. Again, cells orient in the direction of the matrix cords, here at ±45° angles with respect to the gradient. The resulting morphology is a sprout approximately 2 cell diameters thick, notably thicker than the sprouts that develop with strong contact guidance cues aligned in the direction of chemotaxis (Figure 5B,C). Fiber orientation also modulates cell recruitment. When cells elongate and orient in the direction of the VEGF gradient, fewer cells are recruited from the parent vessel and sprout extension is largely due to cell elongation. Compare Figure 5: (A) with no obvious cell orientation 15 cells are recruited, (B) 11 cells are recruited when cells are oriented in the direction of the VEGF gradient, (C) only 3 cells are recruited when cells dramatically elongate, (D) 19 cells are needed when cell orientation is perpendicular to the chemoattractant gradient, and (E) 19 cells are recruited when cells orient at ±45° with respect to the gradient. These results demonstrate the important role of contact guidance and tissue structure in determining cell shape and orientation.

### Changes in average extension rates due to tip cell matrix degradation varies as a function of ECM density

**Figure 6:**
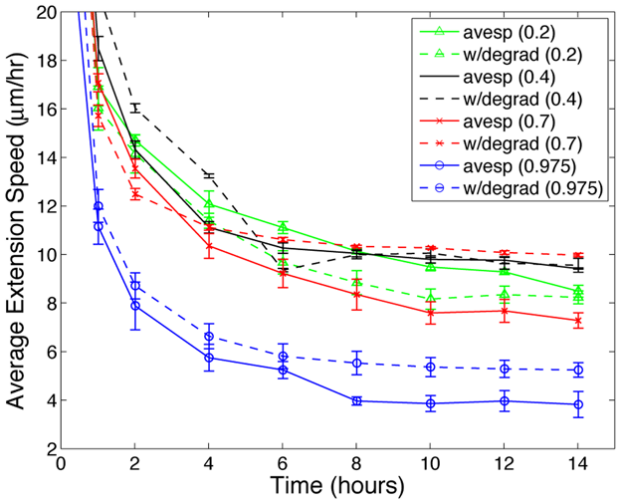
This plot shows that the effect of matrix degradation on average sprout extension speeds depends on the density of the ECM. Solid lines represent average extension speeds without matrix degradation and the corresponding colored dashed lines show average speeds with tip cell matrix degradation. For 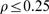, matrix degradation has anti-angiogenic effects. Above 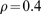, degradation facilitates sprout progression.

**Figure 7:**
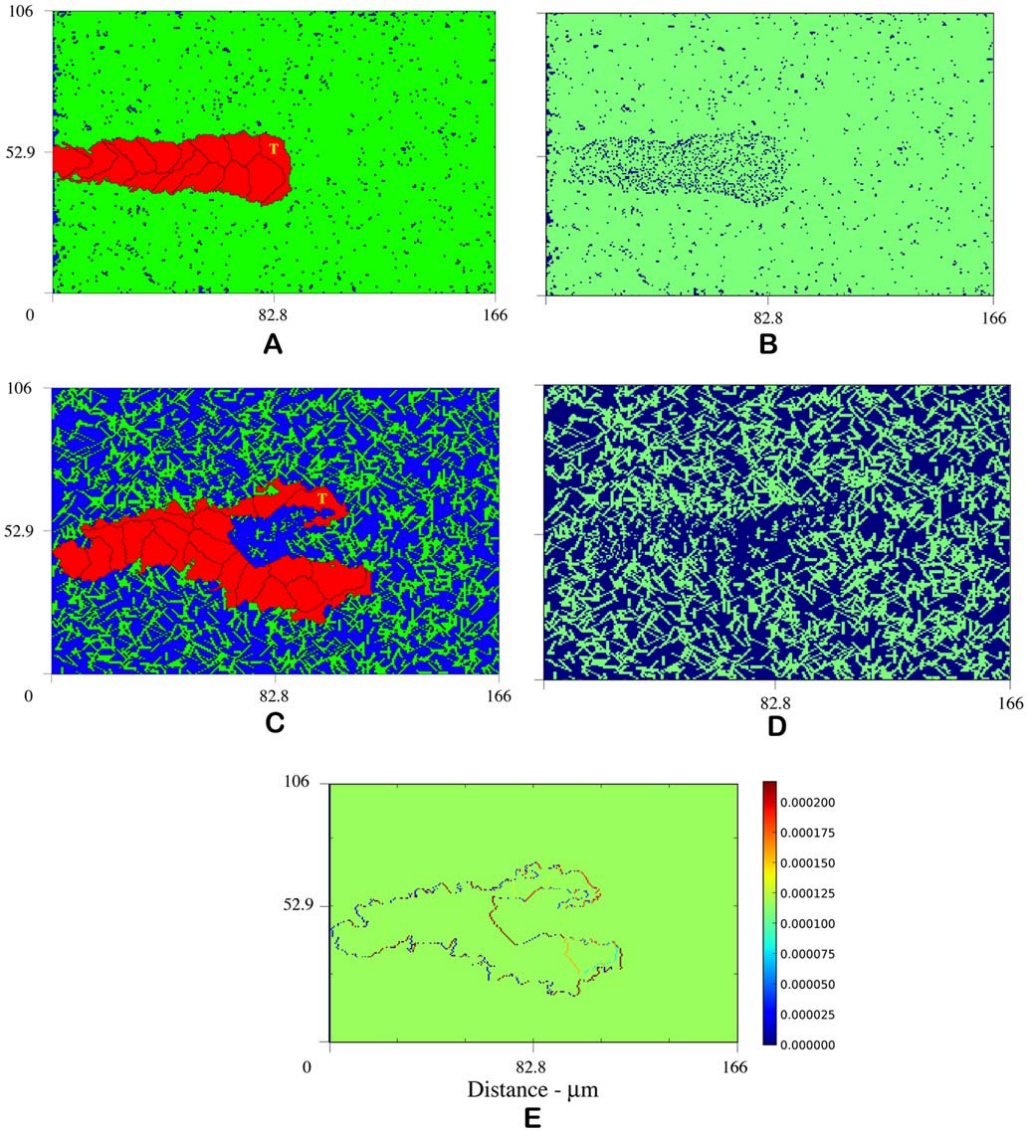
Without degradation, angiogenesis is inhibited at 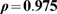 (Figure 3F). (A) shows that tip cell matrix degradation promotes sprout development at 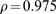 by carving out a path for migration, called a vascular guidance tunnel (B). (C) depicts sprout formation and branching with ECM degradation at 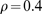, a density not typically conducive to branching, suggesting that high matrix heterogeneity (D) created by tip cell degradation may be a mechanism for branching (Video S3). (E) VEGF gradient profile (pg) shows strong gradient along leading edges of sprout. Snapshots at 14 hours.

During angiogenesis, endothelial cells not only realign matrix fibers, but they also secrete matrix degrading proteases that break down extracellular matrix proteins and facilitate sprout migration through the stroma [20]. To study the effect of matrix degradation on sprout development, we implement matrix degradation by allowing the tip cell to degrade ∼(0.55 µm)^2^ of matrix each minute. We choose this rate of degradation as a rough approximation based on numerical studies of tip cell collagen proteolysis [51]. This rate of degradation is, however, dependent on the availability of ECM, that is, a cell will degrade matrix only if matrix is present. Average sprout extension speeds are recorded and compared with the average extension speeds without matrix degradation for different matrix densities. Figure 6 graphically represents average extension rate pairs for sprouts forming with and without matrix fiber degradation at 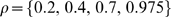 and shows that the effect of matrix degradation depends on matrix density. At 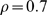 and 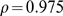, matrix degradation results in approximately a 37% increase in average sprout extension speeds at 14 hours. As matrix fibers are degraded, fewer cell-matrix adhesion sites are bound and therefore cellular attachment is reduced resulting in increased motility. At a matrix density of 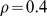, tip cell matrix degradation only seems to have a significant influence on extension speed at earlier times (0–5 hours). This observation suggests that the increase in motility due to a loss of bound focal adhesion sites is limited. On the other hand, Figure 6 also shows that for 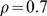, tip cell matrix degradation has the greatest effect at later times, after 10 hours, indicating that at higher densities, a more significant reduction in matrix density is needed before the cluster of cells can realize any noticeable change in sprout progression. Taken together, these results offer some insight into why velocity curves peak at intermediate matrix densities. On more sparse matrices, 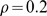, matrix degradation actually slows sprout extension. While this may seem counterintuitive, at lower densities, further reducing fiber density reduces the effectiveness of the ECM to provide a cellular support system that is necessary for normal sprout migration and formation. Thus, depending on the density of the matrix, matrix degradation may result in faster or slower extension speeds, thereby providing pro- and anti-angiogenic effects respectively. Referring to Figure 3F, at 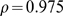 the initial endothelial cell is unable to penetrate the stroma and angiogenesis is completely inhibited. In otherwise identical simulations, however, when the tip cell actively degrades the matrix fibers, the tip cell carves out a path through the ECM and a new vessel sprout develops (Figures 7A,B). This result is empirically validated by very recent experiments from Davis et al. showing that human endothelial cells in extracellular collagen matrices degrade a path through the ECM [52]. This path is referred to as a vascular guidance tunnel and can be seen in Figure 7B.

The effect of degradation is to decrease the density of the ECM and this decrease is entirely localized to the area under and immediately surrounding the sprout body (Figures 7B,D). However, when we vary ECM density systematically as in our numerical experiments (Figure 2A), the reduction is a uniform reduction. Thus, when comparing extensions speeds associated with changes in ECM density from these two different mechanisms (one from degradation that is highly spatially heterogeneous and the other a uniform reduction in ECM density), one must instead calculate the density of the ECM under the sprout and compare sprout velocities at this density. When this is considered, the extension speeds measured when cells degrade the matrix are in agreement with those measured at the corresponding lower ECM density. This subtlety, however, illuminates an important distinction - that sprout development and progression is not independent of the mechanism for matrix reduction (spatially uniform versus localized). Because the velocity curve steepens above 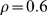, it is quite expected that at these higher densities, any reduction in matrix density will have a significant effect on sprout velocity. Thus, since degradation is very spatially localized mechanism for matrix reduction, the effect of degradation is even more pronounced at higher densities, which is seen in Figure 6.

In our model, without degradation we observe no branching at matrix fiber densities above 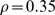. Figures 7C,D show the progress of sprout development at 14 hours with ECM degradation at 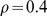 (also see Video S3). A new sprout branches from the primary sprout body, an event that emerges only as a result of featured cellular and molecular level dynamics; no rule specifically incorporating branching is imposed. Tip cell degradation reduces ECM density and sets up very high local anisotropies in the matrix fiber structure (Figure 7D), thereby providing strong contact guidance cues to the developing sprout. Figure 7E shows the VEGF gradient profile in picograms (pg) that was generated during the simulation shown in Figure 7C. This image shows stronger VEGF gradients develop along the leading edges of sprout. Of interest is that even though there is a strong chemotactic incentive at the branching bifurcation, the lack of ECM prohibits sprout progression. These results lend additional support to our hypothesis that high matrix heterogeneities created by tip cell degradation may be a mechanism for sprout branching.

### Sensitivity analysis

To ascertain the variability and sensitivity of our results to the choice of parameters, we vary one parameter at a time, holding fixed all other Table 1 parameters, and record our observations. A summary and explanation of the effects of parameter perturbation can be found in Table 3. Decreasing 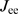 is equivalent to increasing the strength of the bond between endothelial cells. For values of 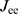 below 10, cell shapes are grossly contorted and unrealistic. For 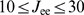, cells elongate to increase their cell-cell contact area. As 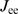 increases, cell-cell adhesion weakens. For 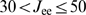, cells move to reduce their surface area contact with each other and are generally rounder in shape. For 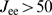, cell-cell adhesion becomes too weak relative to the chemotactic energy acting on the cell, and consequently, the tip cell migrates away from the main sprout. Similarly, lower values of 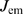 correspond to stronger cell-matrix adhesion energies. For 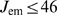, cells are unnaturally distorted in an effort to increase the contact area between the matrix fibers and the cell membrane. At 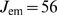, a relatively strong cell-matrix adhesion bond, sprout morphologies are noticeably thicker and more tortuous. Intermediate values (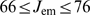) provide a good balance between contact guidance and release of focal adhesion bonds. Sprout morphologies and extension speeds are relatively insensitive to parameter variability within this range. Above 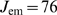, contact guidance is weak. In this case, chemotaxis is the dominant mechanism governing sprout guidance and, consequently, more linear sprouts develop. An extraordinarily large value, 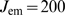, is equivalent to complete inhibition of cell-matrix adhesion, for example by blocking integrin receptors. Thus, at 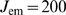, endothelial cells do not adhere to matrix fibers at all and are unable to migrate, even in the presence of chemotatic incentives. The results are insensitive to the binding energies between matrix fibers, 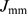, and between interstitial fluid molecules, 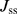, because they are each collectively identified by the same ID and are therefore always like neighbors. Insensitivity is indicated with an “I” in Table 1.

**Table 3:** A summary and explanation of the effects of parameter perturbation in the sensitivity analysis.

	Observation	Parameter Control
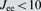	Unrealistic cell shapes	Strong cell-cell bonds
		Deform to increase cell-cell contact
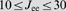	Realistic cell shapes & elongation	Balance between cell-cell contact and motility mechanisms
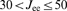	Rounder cells	Deform to decrease cell-cell contact
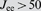	Cells migrate away	Little cell-cell adhesion
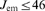	Unrealistic cell shapes	Strong cell-ECM adhesion
		Deform to increase cell-ECM contact
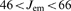	Thicker, tortuous sprouts	Some focal adhesion release but cells “stick” to ECM
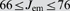	Realistic cell shapes	Balance between contact guidance & release of focal adhesion bonds
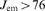	Linear sprouts	Weak contact guidance
		Chemotaxis dominates
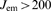	Cells immobile	Inhibition of cell-matrix adhesion
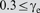	Large cells, fewer recruited	Cells easily deviate from target size
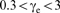	Realistic cell sizes	Balance between growth & chemotaxis
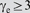	Tip cell detachment	Pressure to keep stalk cell size>chemotactic energy of stalk cells
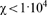	No migration	Chemotactic stimulus too weak
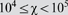	Slow migration	Chemotactic stimulus too weak
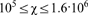	Cells migrate faster with increasing 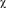	Balance between chemotaxis, growth, and contact guidance
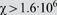	Cells are pulled apart	Chemotactic stimulus too strong
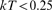	Cells dissociate	Moderated by continuity constraint
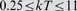	Faster sprout migration with increasing kT	Lowers probability that large change to total energy is accepted

In addition, the results do not depend on the compressibility properties of the matrix fibers or interstitial fluid, 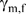, since the total mass of these ECM components is conserved. We vary 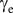 between 0.3 and 3. Decreasing 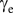 makes it easier for the cells to deviate from their target volume. Therefore, at 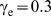, the cells grow to a larger size overall, and consequently, fewer cells are recruited from the parent vessel. However, average extension speeds are not affected. That sprouts are able to maintain their average velocity with fewer recruited cells highlights cell growth as another mechanism for sprout extension. On the other hand, increasing 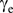 produces smaller cells, and in this case, more cells are recruited. At 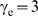, the tip cell migrates away from the main body of the sprout. This is because of the chemotactic sensitivity differential between the tip cell and the stalk cells. Here the relative pressure on a cell to maintain its target volume (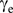) is greater than the chemotactic energy of the stalk cells, but not greater than the chemotactic incentives for the tip cell. Thus the tip cell detaches.

**Figure 8:**
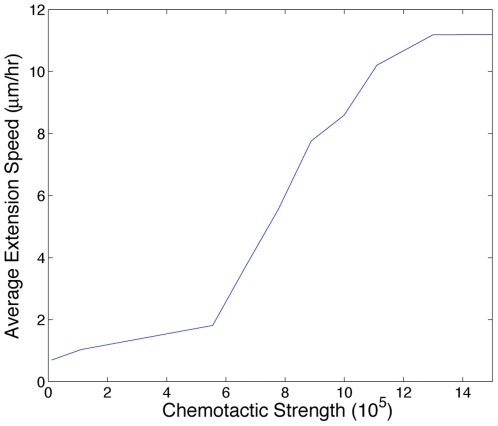
Plot showing the effect of varying the chemotactic sensitivity parameter, 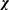, on average sprout extension speed at 14 hours. Below 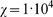, chemotactic cues are not strong enough relative to the energies associated with adhesion and growth to induce motility. Above 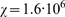, chemotactic incentives are so strong relative to adhesion and growth that the cells dissociate.

Figure 8 shows how the average extension speed of a sprout varies with increasing chemotactic sensitivity,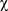. Average speeds are calculated at 14 hours. Above 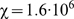, the physical integrity of the endothelial cells is compromised and cells dissociate due to the relatively strong chemotactic stimulus. Below 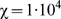, chemotactic gradients provide insufficient migratory cues relative to the adhesion energies and the growth constraint, and consequently, the initial cell does not migrate into the stroma. At intermediate values (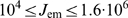), sprouts migrate faster with increasing 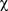, but sprout morphologies are unaffected. To determine the effect of changing the probability that energetically unfavorable events occur, we vary the parameter 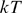, where 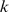 is the Boltzmann constant and 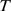 is the effective temperature that corresponds to the amplitude of cell membrane fluctuations. Increasing 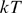 decreases the probability that an update adding to system energy will be accepted. Increasing 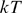 effects faster average sprout extension speeds, but no noticeable changes in cell shape, the number of cells recruited, or sprout morphology. This parameter becomes insensitive as it decreases because it is moderated by the continuity constraint. For example, as the probability to accept a change that increases system energy goes up (decreasing 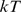), we would expect cells to break up easily, but in this case, the continuity constraint circumvents this effect.

## Discussion

The extracellular matrix has attracted a great deal of attention from researchers and experimentalists because of its vital role as a modulator of morphogenic processes. Inspired by our previous finding that the stromal heterogeneity has a strong influence on sprout morphogenesis [19], in this work, we focus on one particular aspect of the biophysical properties of the stromal environment, the topography of ECM. Identifying and elucidating the mechanisms through which the ECM contributes to changes in cell shape and function is of critical importance to many morphogenic events, including angiogenesis, wound healing, embryogenesis, and tumor invasion. We use a two-dimensional cell-based model of angiogenesis as a framework to explore the effects of ECM topography on cell-cell and cell-matrix dynamics. Our modeling approach captures the morphology of the cells and of emergent multi-cellular structures, which allows a quantitative description of physical characteristics at the cellular level, such as cell shape, cell orientation, and sprout thickness. By adjusting key parameters in our model, we are able to simulate: (i) a frequent observation in embryogenesis whereby cells use cell-cell adhesion to rapidly traverse along the sprout, (ii) single cell migration as seen in fibroblasts during wound healing, and (iii) different cell shapes indicative of different cell phenotypes or species. Our results indicate that the density or connectedness of the matrix, local proteolytic matrix degradation, and fiber alignment affect sprout extension speeds. We record peak migration speeds in tissues that have a similar collagen content to that seen in the cornea. We observe density dependent pro- and anti-angiogenic effects and propose that high matrix fiber anisotropy provides strong contact guidance cues and is a mechanism for initiating sprout branching. Finally, we provide strong evidence that contact guidance influences cell orientation by examining sprout development on engineered matrix patterns.

During morphogenesis, cells actively restructure and condition the extracellular matrix for migration through proteolytic degradation and fiber reorganization and alignment [9]. Our studies suggest that contact guidance cues are mediated by changes in matrix fiber density and isotropy, network connectedness, and fiber orientation. These findings collectively support the hypothesis that contact guidance cues play a major role in determining sprout morphology and the average rate of capillary sprout extension. Our results strongly suggest that the contact guidance cues established through high matrix fiber inhomogeneity in the stroma may be a mechanism for sprout branching. Applying our results in the context of tumor-induced angiogenesis, local changes in ECM density that create matrix anisotropies, in concert with fiber alignment, may contribute to the accelerated extension speeds reported as sprouts approach the tumor. In addition, fiber density is not constant in the extratumoral environment. The density of the extracellular matrix is lower near the tumor due to the secretion of matrix degrading proteases by tumor cells. If these lower regions of matrix density are within the range we predict to be conducive to branching, this could help explain why an increase in branching, known as the brush border effect, is seen *in vivo* as sprouts get close to the tumor.

Describing matrix fiber cross-linking, viscous interstitial flow, and cell-matrix interactions dynamically within the same modeling framework is currently one of the big challenges in modeling morphological events. A first step is to provide an explicit description of the ECM and cell-matrix interactions, which we have done in this model. Our model is one of few to provide an explicit treatment of the ECM and the only to do so in a cell-based framework. Our model incorporates some key cell-ECM interactions, including adhesion and degradation. We do not, however, consider matrix reorganization or remodeling that can result from endothelial cell matrix secretion, adhesion, and migration. Nor do we consider dynamic matrix fiber cross-linking, which would allow an explicit description of matrix stiffness and the ability to quantify the effects of substrate rigidity on cellular behavior. Instead, we employ a static ECM in this initial investigation so that we can confidently associate vascular morphology with extracellular matrix topology. By doing so, we have shown that matrix topology alone is enough to regulate cell shape and orientation and to initiate sprout branching. Dynamic imaging techniques have recently been developed and are now being used in *in vivo* embryogenesis systems to describe ECM macroassembly dynamics [1] that will facilitate further advances in modeling matrix mechanics.

It is worth pointing out that at a distance of 100 µm from a tumor 1 mm in diameter, we specify a linear source of VEGF. This choice ensures little or no gradient in the transverse or y–direction and allows us to attribute lateral cell and sprout movement to the mechanical effects of the matrix. Different spatial profiles of VEGF, for example a parabolic source or local sinks and sources of VEGF in the ECM, could also contribute to branching and varied morphological patterns. The effect of different VEGF profiles on angiogenesis has been theoretically modeled by Anderson and Chaplain [53]. The VEGF profile would also be altered by variations in the density of the matrix. Changes in matrix density also affect VEGF diffusivity and binding to the matrix, and therefore can influence local VEGF gradients. However, since we assume a constant diffusivity coefficient and do not yet consider VEGF bound isoforms in our model, in these numerical experiments the VEGF concentration profile does not adjust with the variation in matrix density. These assumptions allow us to attribute any observed differences in extension speed and morphology directly to changes in matrix density. In our previous work [19], our model predicts markedly different vascular morphologies coincident with changes in the VEGF gradients. We expect that the changes in VEGF gradients that may result from modifications to matrix density could also have significant consequences to sprout velocity and morphology. In particular, matrix-induced changes in VEGF gradients may explain reported increases in sprout velocity as the sprout approaches a tumor.

### Clinical implications: ECM targeted angiogenic therapies

Increased understanding leading to the ability to control angiogenesis *in vivo* has serious clinical implications. Angiogenesis is a crucial event to many physiological processes. Embryonic development and endometrium vascularization, arteriogenesis resulting from ischemia and vessel occlusion, wound healing and tissue repair are all homeostatic processes that require new vessel growth for normal function. Angiogenesis can also lead to pathological conditions. Tumor angiogenesis, proliferative diabetic retinopathy and macular degeneration, psoriasis and rheumatoid arthritis occur when angiogenesis is unhalted [54]. On the other hand, insufficient vessel growth can lead to heart attack, stroke, and impaired ulcer and wound healing. Existing angiogenic therapies can be broadly categorized as those that (1) target growth factors or growth factor cell receptors that stimulate vessel growth, (2) block cell invasion into the stroma, and (3) directly induce endothelial cell apoptosis. Because of its established prominence in both homeostatic and aberrant angiogenesis, VEGF and its receptors are prime therapeutic targets. There is overwhelming experimental evidence that in order to form functional vessels, the various VEGF isoforms must be precisely regulated and that the blockage of even a single growth factor might limit tumor-induced vascular growth [20],[23],[55]. The most promising approaches to anti-angiogenesis therapies are those based on blocking VEGF or VEGF receptors [56]. VEGF neutralizing antibodies, soluble VEGF receptors, and receptor tyrosine kinase inhibitors are examples of therapies currently being utilized or that are undergoing clinical trials [57]. One problem associated with targeting growth factors as therapeutic agents is that they are often constitutively expressed *in vivo* and can be proteolytically released. Thus tight control is, in practice, hard to maintain. For example, it is known that connective tissue, which contains some of the same fibrous proteins that are found in the ECM, can significantly inhibit cell migration and prevent the formation of sprouts [20].

The ECM and cell-matrix associations also provide promising possibilities for angiotherapy, but have only more recently received attention as targets and are in less advanced stages of clinical development. Consequently, modeling and simulation have the potential to contribute to and propel further advancement. Current therapeutic interventions aimed at cell-matrix interactions during angiogenesis focus on tissue inhibitors of metalloproteinases (TIMPs) and on integrin-mediated cellular adhesion [54]. Blocking proteolysis is intended to inhibit cellular migration into the stroma and to prohibit MMP-dependent release and activation of ECM sequestered angiogenic factors. Our results indicate that regulating the cellular production of matrix degrading proteases can shift sprout velocity curves for the purpose of promoting or inhibiting angiogenesis. We show that at low matrix densities (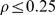), matrix degradation has anti-angiogenic effects, whereas above 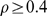, degradation facilitates sprout progression.

Using our model, we regulate cell-matrix binding affinity (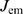) and control the number of focal adhesion binding sites available in the ECM (density modulation) to test the efficacy of integrin specific anti-angiogenic therapies. The 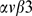 integrin receptor is significantly upregulated in angiogenic vessels compared to mature vessels [54], making this receptor one logical therapeutic choice. Setting 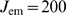 is equivalent to blocking integrin receptors. Our simulations show that decreasing the binding affinity of integrin receptors prevents endothelial cells from adhering to matrix fibers and cells are unable to migrate even in the presence of chemotactic incentives. We also show that cellular motility is inhibited at high matrix densities. This is due to the greater number of focal adhesion binding sites available. Simulations suggest that regulating the affinity or number of cell-matrix focal adhesion sites either biochemically or mechanically produces anti-angiogenic effects.

In these collective studies, we use the model to isolate and examine variations in fiber density and structure, and proteolytic matrix degradation as independent mechanisms that control vascular morphogenesis. Nonetheless, the integrin, protease, and growth factors systems are highly connected and provide regulatory feedback for each other [54]. Thus, there is still a need for more in depth investigations of the relationship between extracellular stimuli and cellular function. In particular, studies focusing on intracellular signaling and cross-talk between the integrin and growth factor receptors are of key importance. In addition, experimental models are needed to measure critical biochemical activity, for example, matrix proteolysis during angiogenesis, and to verify the predictions made herein regarding the pro- and anti-angiogenic effects of manipulating the ECM.

## Supporting Information

Figure S1 Cell elongation. For a different parameter set, fewer cells are recruited from the parent vessel and cells elongate. Here cells are approximately 40 µm in length and the average extension speed at 14 hours is 6.8 µm/hr. J_{ee,em,ef}_ = {42,76,66}, χ_tip_ = 1.55 χ, χ_{stalk, prolif}_ = 1.45 χ.
(2.30 MB TIF)

Figure S2 Our model accurately captures the cellular response to topographical guidance (no VEGF) on patterned substratum. Compare this image with morphological data of fibroblasts stained for actin and tubulin showing that cells alter their shape, orientation, and polarity to align with the direction of the grooves [see Figure 5e,f from Oakley *et al.* 1997]. Simulation is on similarly patterned substrate and demonstrates the flexibility of our model to capture a variety of different morphological phenomena.
(0.47 MB TIF)

Video S1 Single cell migration/invasion
(4.83 MB MOV)

Video S2 Preferential migration along stretched cells
(3.19 MB MP4)

Video S3 Matrix anisotropy induces branching
(5.05 MB MP4)
