# Supplementary figures and images for: Topography of Extracellular Matrix Mediates Vascular Morphogenesis and Migration Speeds in Angiogenesis
Source: PLoS Comput Biol. 2009 Jul 24;5(7):e1000445. doi: 10.1371/journal.pcbi.1000445 (PMC2709079; doi:10.1371/journal.pcbi.1000445)

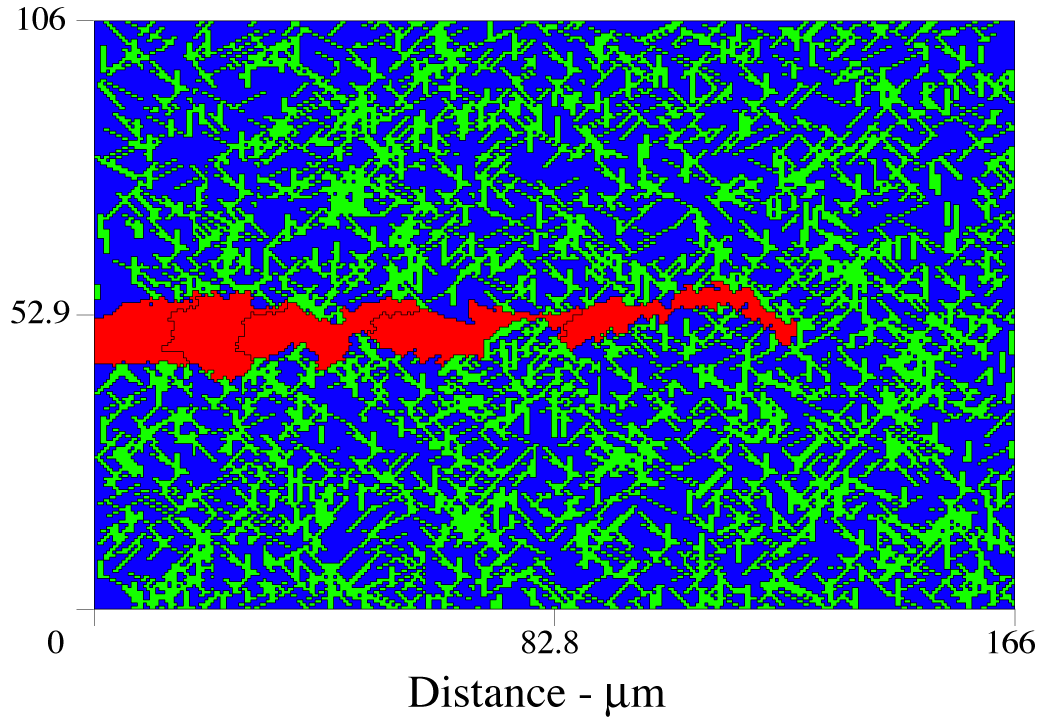

Supplement: Figure S1 — Cell elongation. For a different parameter set, fewer cells are recruited from the parent vessel and cells elongate. Here cells are approximately 40 µm in length and the average extension speed at 14 hours is 6.8 µm/hr. J{ee,em,ef} = {42,76,66}, χtip = 1.55 χ, χ{stalk, prolif} = 1.45 χ. (2.30 MB TIF) [file pcbi.1000445.s001.tif]

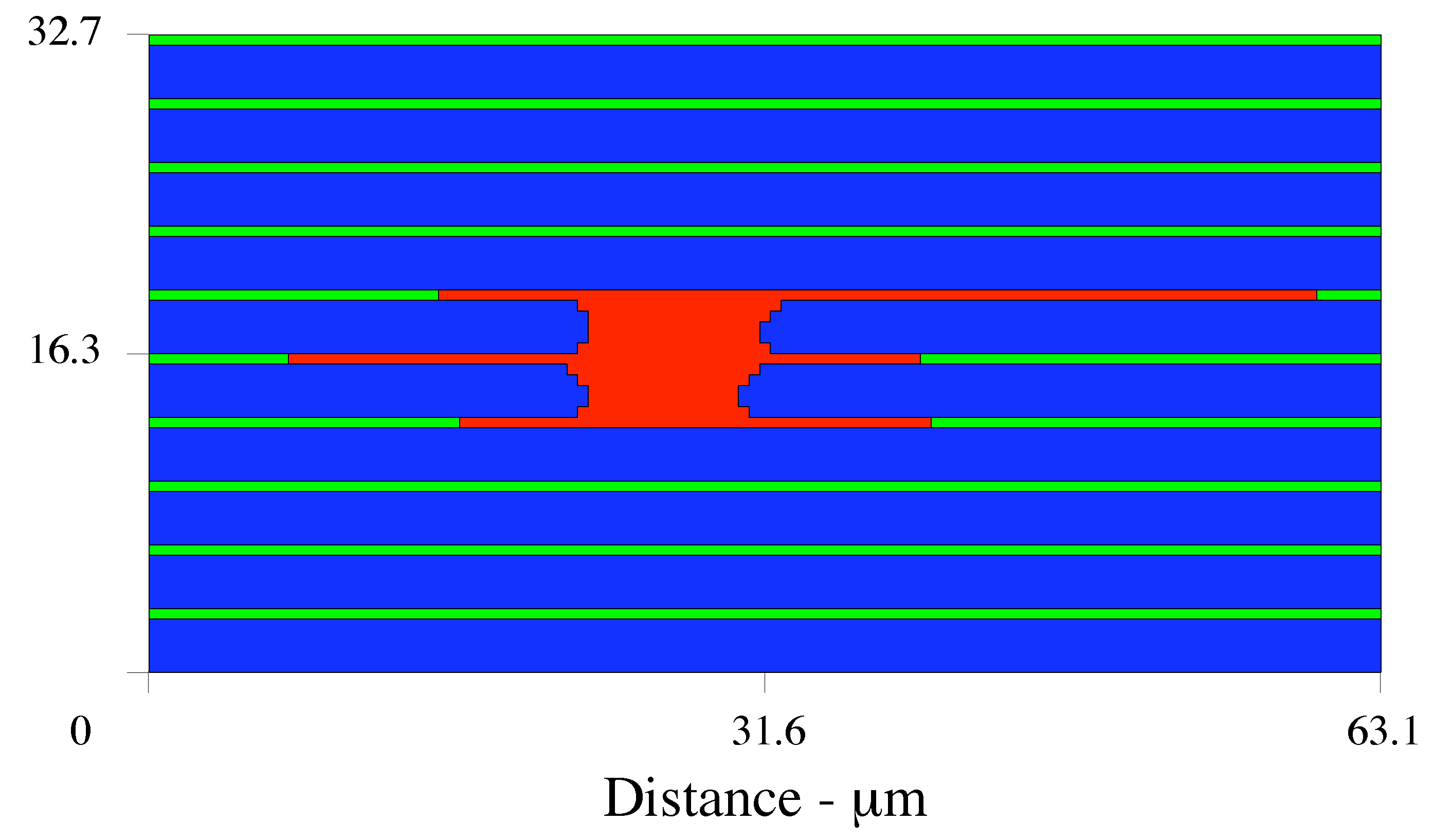

Supplement: Figure S2 — Our model accurately captures the cellular response to topographical guidance (no VEGF) on patterned substratum. Compare this image with morphological data of fibroblasts stained for actin and tubulin showing that cells alter their shape, orientation, and polarity to align with the direction of the grooves [see Figure 5e,f from Oakley et al. 1997]. Simulation is on similarly patterned substrate and demonstrates the flexibility of our model to capture a variety of different morphological phenomena. (0.47 MB TIF) [file pcbi.1000445.s002.tif]
